# Maternal Mortality in the Governorate of Tunis between 2017 and 2023: evolution of Maternal Mortality Ratio and the main causes of death

**DOI:** 10.12688/f1000research.155009.1

**Published:** 2024-10-11

**Authors:** khaoula magdoud, hanene rezigui, Nejla Arifi, Sirine Bayar, Hamza Ben Abdallah, Hassine Saber Abouda, Rim Ben Hmid

**Affiliations:** 1 University Tunis El Manar Tunisia, Faculty of medicine of Tunis, Tunis, Tunisia; 2 Maternity and Neonatology Center of Tunis, Tunis, Tunisia; 3 Regional Health Directorate Of Tunis, Tunis, Tunisia

**Keywords:** Maternal mortality, Causes, Hemorrhage, COVID-19, Postpartum period

## Abstract

**Background:**

Maternal mortality is the main indicator of maternal health worldwide. The aims of your study were to determine the Maternal Mortality Ratio (MMR) in the governorate of Tunis and to identify the main causes of maternal death.

**Methods:**

We included all maternal deaths between January 2017 and December 2023, reported to the Tunis Regional Health Directorate. The data collected included the MMR per 100000 live birth, sociodemographic characteristics, causes of death, circumstances of death, autopsy findings, and preventability.

**Results:**

Sixty one maternal deaths were recorded. The average of MMR was 46, 88 per 100000 live births. Two peaks in the MMR were noted in 2017 and 2020. The average age of the patients was 34, 1 years (±5.1). We noted that 43, 75 % of the patients were not residents of the governorate of Tunis. The postpartum period was the most critical. In fact, 83, 33 % of maternal deaths occurred postpartum. Hemorrhage was the main etiology in 20.8% of cases. The second cause was COVID- 19complicationsin 16, 6% of cases.

**Conclusion:**

This study of maternal mortality in Tunis opens the debate on the effectiveness of maternal health policies in Tunisia and the areas for improvement.

## Introduction

Maternal mortality is a major public health problem. It is defined by the World Health Organization (WHO) as: “Regardless of the length or location of the pregnancy, maternal mortality is defined as the death of a woman while she is pregnant or within 42 days after the pregnancy’s termination from any cause connected to or aggravated by the pregnancy or its management, but not from unintentional or incidental causes.” ^1^

According to WHO data for 2020, maternal mortality is reaching unprecedented levels. Around 287,000 women died during or after pregnancy or childbirth; almost 95% of maternal deaths occurred in low- and middle-income countries. ^2^

The maternal mortality ratio (MMR) in Tunisia has improved from 62 in 2000 to 37 in 2020. ^1^ The situation in Tunisia shows that maternal mortality has decreased, but insufficiently in relation to the level of effort required and the objectives set. ^3^

Among the direct causes of maternal death are three main pathologies: Hemorrhage, infectious complications and complications linked to pregnancy toxemia. Their frequency and severity reflect the level of development and organization of the healthcare and information system. ^3^

Maternal death is usually avoidable in 80% of cases, even in situations where countries have few resources. ^3^

Hence, the aims of your study are to determine the MMR in the Governorate of Tunis and to identify the main causes of maternal death.

## Methods

### Study design

This was a retrospective descriptive study focusing on the records of maternal deaths that occurred in public health facilities and were reported to the Regional Health Directorate of Tunis.

### Setting

In June 2024, information on maternal death records in the governorate of Tunis from January 2017 to December 2023was collected.

Tunis is the capital of Tunisia. The population of Tunis was 1,078,412 in January 2023, which represents 8, 8% of the general population. ^4^

### Participants

Inclusion Criteria: We included all deaths of women that occurred during pregnancy or within 42 days after its termination in public health facilities.

Non-Inclusion Criteria: We did not include maternal deaths that occurred due to an accident or trauma, and cases where the records lacked data regarding the circumstances of the death.

### Variables

After obtaining approval from the Director of the Regional Health Directorate of Tunis, we collected data from patients’ medical records, the regional committee’s maternal death follow-up reports, and autopsy reports if performed. Indeed, since 1999, Tunisia has set up regional and national committee’s maternal deathfollow-up. ^5^ The main roles of these committees are to determine the cause of death and to propose preventive measures.

The data collected included the MMR per 100000LB, sociodemographic characteristics (age, marital status, place of residence), causes of death, circumstances of death (mode of delivery, time of death, place), autopsy findings, and preventability.

The causes of death are subdivided into direct and indirect causes: ^1^

• Death from direct obstetric cause: these are those resulting from obstetric complications (pregnancy, labor, and postpartum period), interventions, omissions, incorrect treatment, or a chain of events resulting from any of the above factors.
• Death from indirect cause: these are those resulting from a pre-existing disease or a condition that developed during pregnancy without being due to direct obstetric causes but was aggravated by the physiological effects of pregnancy.

### Statistical methods

For the entry, analysis, and processing of the collected data, we used Microsoft Office Excel, is available for download at https://www.office.com/?omkt=fr-FR.

Qualitative variables will be described in terms of frequencies and percentages, and quantitative variables in terms of means and standard deviations.

## Results

During the seven-year period from January 2017 to December 2023, 61 maternal deaths were recorded ( Table 1). In 2017 and 2020, we recorded the highest number of maternal deaths with 13 and 12 cases, respectively.

**Table 1.** The maternal mortality ratio in Tunis between 2017 and 2023.

Year	Live births	Number of maternal death	MMR
**2017**	17352	13	74.9
**2018**	20918	8	38.24
**2019**	18825	7	37.18
**2020**	16846	12	71.23
**2021**	15010	7	46.63
**2022**	14162	7	49.42
**2023**	26986	7	25.93
**Total**	130099	61	**46.88**

**Figure 1.**
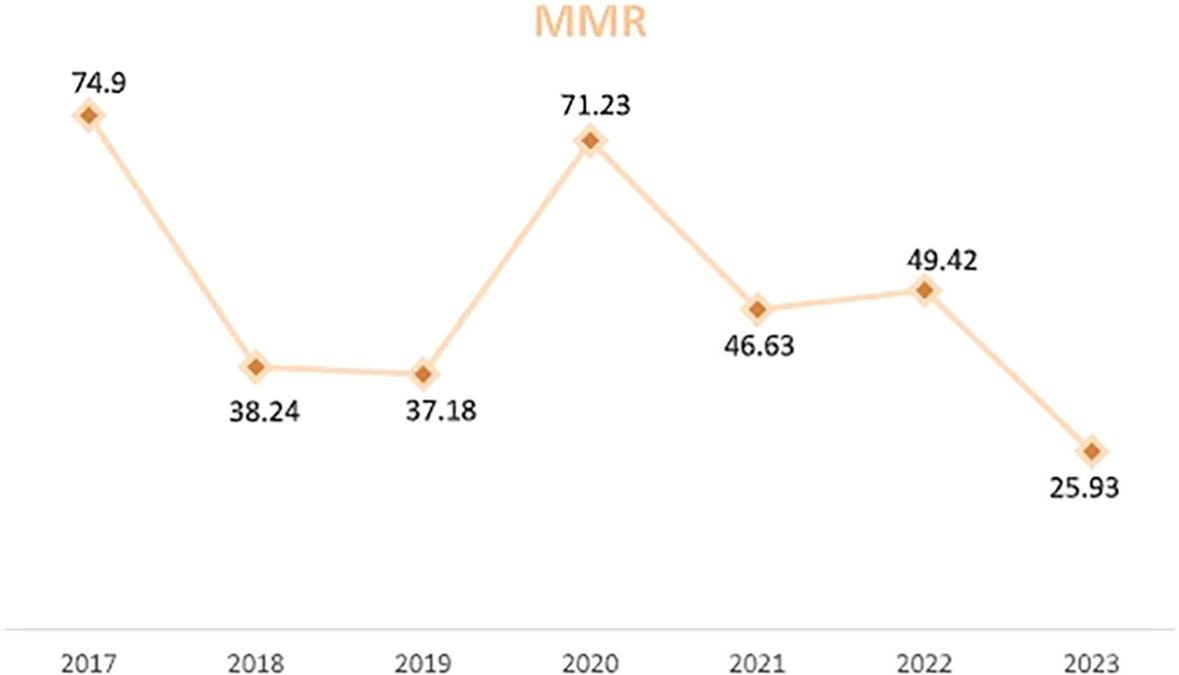
Evolution of the maternal mortality ratio (MMR) in Tunis between 2017 and 2023.

The average MMR was 46, 88 per 100000 LB. The evolution of MMR was marked by two peaks in 2017 and 2020 and a decrease in MMR of 34, 57% between 2017 and 2023 ( Figure 1).

### Characteristics of the population ( Table 2)

**Table 2.** Sociodemographic and clinical characteristics of maternal deaths in Tunis between 2017 and 2023.

data	Average/number (n=48)	Percentage (%)
**Age**		
- Average age	34.1 years (±5.1)	
- Age less than 35 years	30	62,5
- Age greater ≥35 years	18	37,5
**Marital status**		
- Married	48	
- Single	0	
**Place of residence**		
- Tunis	27	56,25
- Other governorates	21	43,75
**Medical history**		
- No history	40	83,33
- Heart disease	3	6,25
- Hypothyroidism	1	2,08
- Sickle cell disease	1	2,08
- Asthma	1	2,08
- HBP	1	2,08
**Obstetric history**		
- Average parity	2.02	
- Number of children ≥ 2	34	70,83
- History of cesarean section	19	39,68
- History of ectopic pregnancy	1	2,08
- Miscarriage	8	16,66
- Voluntary termination of pregnancy	1	2,08
**Last pregnancy**		
- Monitored/Not monitored	29/19	60,41/39,58
- No complications	35	72,91
- HBP	5	10,41
- Gestational diabetes	5	10,41
- Premature rupture of membranes	2	4,16
- Premature birth	1	2,08

During the study period, 61 maternal deaths were recorded, but 13 records lacked data related to the circumstances of the death. Thus, this work focused on 48 records of maternal deaths.

The average age of the patients was 34, 1 years (±5.1), with extremes ranging from 20 to 46years. In 47, 9% of cases, the deceased women were aged between 30 and 34 years. All women were married. We found that 56, 75 % (n=27) resided in Tunis, and 43,75% (n=21) of the patients were not residents of the governorate of Tunis.

A percentage of 83.33% (n=40) of the women had no medical history. Heart disease was the most reported pathology, found in 6.25% (n=3) of studied women.

The average parity of the patients was 2, 02 with extremes from 0 to 5. We noted that 70, 83% (n=34) of the deceased women had two or more children. A history of cesarean delivery was noted in 39, 58% of cases (n=19).

Sixty percent (n=29) of the deceased women had received proper follow-up during their last pregnancy. We noted that 87, 5 % (n=35) of the deceased women had no pathology during the last pregnancy.

### Data related to maternal deaths ( Table 3)

**Table 3.** Data related to maternal deaths.

Data	Number (n=48)	Percentage (%)
**Time of death**		
- During pregnancy	5	10,41
- During childbirth	1	2,08
- Post abortion	2	4,16
- Postpartum	40	83,33
**Place of maternal death**		
- Intensive care unit	33	68,75
- Maternity ward	15	31,25
**Causes of maternal death**		
**Causes directs**	**30**	**62,5**
- Hemorrhage	10	20,83
- Pre eclampsia	5	10,41
- Acute hepatic steatosis	5	10,41
- Sepsie	4	8,33
- Pulmonary embolism	3	6,25
- Anesthesia factors	3	6,25
**Causes indirects**	**17**	**35,41**
- SARS-COV-2 infection	8	16,66
- H1N1 infection	3	6,25
- Meningitis	1	2,08
- Heart attack	2	4,16
- Sickle cell crisis	1	2,08
- Peritonitis	2	4,16
**unknown**	**1**	**2,08**

Time of Death: we noted that 83, 33% (n=40) of maternal deaths occurred postpartum, of which 80 % of cases (n=32) were within 24 hours.

Mode of Delivery: For women who died in postpartum (n=40), delivery was by cesarean section in 87, 5% (n=35) of cases.

Place of Maternal Death: Sixty-nine percent of women (n=33) died in an intensive care unit, and 31, 25% (n=15) died in a maternity ward.

**Figure 2.**
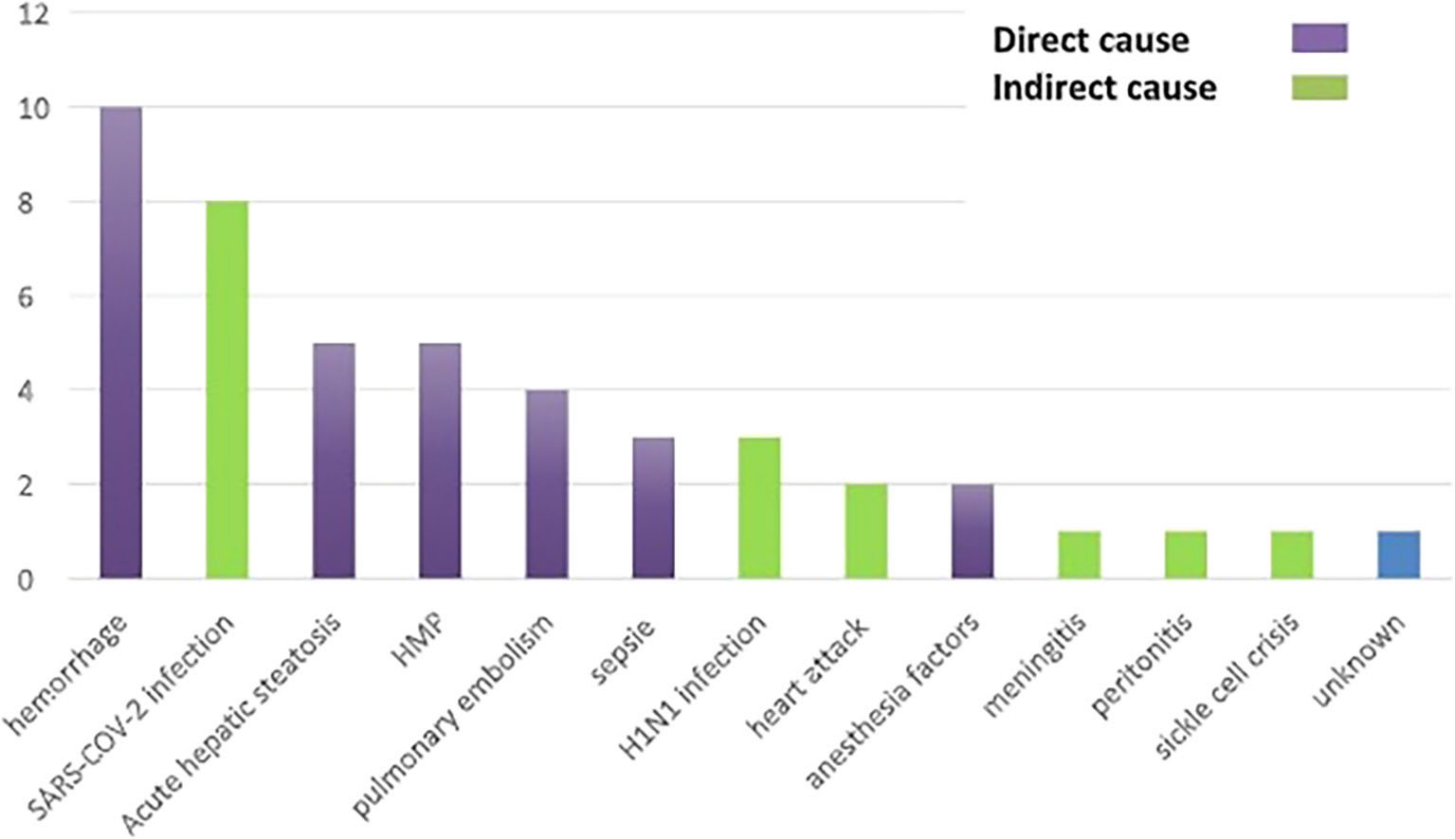
The causes of maternal deaths in Tunis between 2017 and 2023.

Causes of Maternal Death: The causes of maternal death were direct in 62.5% of cases (n=30) and indirect in 35, 4% of cases (n=17). The cause of one maternal death was not identified ( Figure 2).

Direct Causes: Hemorrhage was the main etiology in 20, 8% of cases (n=10). Preeclampsia and acute fatty liver of pregnancy each accounted for 10.4% of the causes of death (n=5).

Indirect Causes: Indirect causes accounted for 35, 4% (n=17) of the causes of death. A percentage of 16, 6% (n=8) of deaths were related to complications from COVID-19 infection, 6, 25% (n=3) from H1N1 infection, 4, 16% (n=2) following a myocardial infarction, 2% (n=1) peritonitis, 2% (n=1) meningitis of ENT origin, and 2% (n=1) sickle cell crisis.

Autopsy: Autopsy was performed in 8, 5% of cases (n=4). We had only two autopsy results: The first was a woman who died from an anesthetic accident, and the second from a massive pulmonary embolism.

Preventability: In our study, the maternal death follow-up committee deemed the death preventable in 68, 8% of cases (n=33).

## Discussion

### The maternal mortality rate and its evolution

MMR is the primary indicator of maternal health worldwide. In our study, the average MMR between 2017 and 2023 in the Tunis governorate was 46, 88/100 000 LB. Our study observed two peaks in maternal mortality, with 13 maternal deaths in 2017 and 12 maternal deaths in 2020. Despite the peak in 2020, there was a 34, 57 % drop in MMR between 2017 and 2023

In line with the Millennium Development Goals (MDGs), the objectives were to reduce maternal mortality by three-quarters (MDG 5) and under-five mortality by two-thirds (MDG 4). ^6^ Although this objective has been achieved for infant mortality, the objective for reducing maternal mortality has not been met. ^3^

Indeed, following the 2011 Tunisian Revolution, the country faced numerous challenges, including democratic transition, economic difficulties, and demographic shifts.

According to the Tunisian Ministry of Health’s report, one of the main factors contributing to maternal deaths appears to be the dysfunction of the healthcare system, including delays in making the decision to seek medical care and delays in referral to an appropriate healthcare facility. ^3^

According to our findings, 43, 75% (n=21) of the patients were not residents of the governorate of Tunis. Most often, these patients are transferred from other governorates especially the northwest governorates. This explains the delay in transferring patients to an appropriate maternity hospital and, consequently, the delay in care. Indeed, in Tunisia, as in other countries, the disparity in access to care between inland and coastal regions is a public health problem.

According to 2019 data from the Tunisian Ministry of Health, in Greater Tunis (the four governorates: Tunis, Ariana, Ben Arous, and Manouba), there are 4.92 gynecologists-obstetricians per 10,000 women of reproductive age, while in the Northwest regions (the four governorates: Jendouba, Kef, Siliana, and Beja), there are only 1.21 gynecologists-obstetricians per 10,000 women of reproductive age. ^7^ Similarly, in terms of facilities, all maternity hospitals with intensive care units for adults and newborns are in coastal regions.

On a global scale, a significant disparity in mortality rates between developed and developing countries has been noticed. ^8^ Indeed, according to WHO data from 2020, 99% of maternal deaths occur in developing countries. ^9^

In Africa, the average MMR is 415/100000 LB. ^7^ This rate differs significantly by country, ranging from 37/100000 LB in Egypt to 1,150/100000 LB in South Sudan. ^8^ In Tunisia, the situation is intermediate with a MMR at 37/100000LB in 2020. ^1^

### The causes of maternal death

Since the WHO implemented the ICD-10 for deaths during pregnancy, childbirth and the postpartum period in 2012, the collection, analysis and interpretation of maternal mortality data worldwide has been standardized. ^10– 12^

There are two main causes of maternal death: direct and indirect. Direct causes are the most frequent according to literature data. ^13– 17^

Similarly, in our study, direct causes accounted for 62, 5% of maternal deaths. Hemorrhage was the leading direct cause of death in 20, 8% of cases, followed by complications of hypertensive disorders in 10,4% of cases.

This aligns with the WHO’s findings that approximately three-quarters of all maternal deaths globally are direct, caused by hemorrhage, infection, hypertension during pregnancy, and unsafe abortion. ^18^

In developing countries, hemorrhage remains the leading cause of maternal mortality, with rates ranging from 28.8% to 43.4%. ^17, 19, 20^

Preeclampsia is a common complication of pregnancy, affecting 2-8% of pregnancies. ^21^ It can lead to serious maternal health consequences, including eclampsia, haemostasis disorders, HELLP syndrome, kidney failure, and retroplacental hematoma. ^22^

It is a major direct cause of maternal mortality in both developing and developed countries. ^17, 18, 23, 24^

Developed countries like the United States and China have witnessed a significant evolution in the causes of maternal mortality. There has been a substantial decline in direct causes, while indirect causes, particularly cardiovascular complications, are increasing. ^23, 24^

In our study, indirect causes accounted for 43% of maternal deaths, with COVID-19 infection being the most common indirect cause, responsible for 8 deaths. This explains the peak in MMR in 2020. Complications of COVID-19 infection were the second leading cause of mortality.

Indeed, pregnant women are considered vulnerable to viral pneumonias, including COVID-19 infection. ^25^

COVID-19 attacks ciliated epithelial cells via the angiotensin-converting enzyme 2 receptor. This receptor is expressed in the cardiovascular, intestinal, pulmonary and renal systems, as well as in the placenta and fetal tissues. Clinical manifestations are due to direct attack on target cells and host response. ^26^

The impact of COVID-19 in pregnant women has been the subject of several studies, but the results are controversial.

Some studies have shown no increase in mortality risk for pregnant women infected with COVID-19. ^27– 29^

However, a Brazilian study published in 2023 found an increased risk of morbidity and mortality among pregnant women infected with COVID-19 compared to a control group. Factors associated with maternal mortality in COVID-19 infected women were cesarean delivery, third-trimester infection, and comorbidities. ^30^ Vaccination was a protective factor. ^30^ Similarly, a meta-analysis involving 11 studies and 13,136 pregnant women found that COVID-19 infection increased maternal mortality regardless of the term of pregnancy. ^31^

According to the WHO, during the COVID-19 pandemic, maternal mortality was likely influenced by two mechanisms: deaths due to the direct interaction between pregnancy and COVID-19, and those caused by complications of pregnancy that went untreated due to disruptions in healthcare services. ^2^

The main factors associated with maternal death according to the literature data were advanced maternal age, non-follow-up of the pregnancy and delivery by cesarean section. ^24, 32^

In our series, 87, 5% of patients who died postpartum had delivered by cesarean section.

For years in Tunisia, the rate of cesarean delivery has been increasing with a rate of 43, 2 % in 2018 (46,4 % in urban population). ^33^

Certainly, cesarean section has improved maternal and neonatal prognosis but at high rates, it can be a factor that increases maternal morbidity and mortality. ^34, 35^

Pending the results of the implementation of the national maternal and neonatal health strategy 2020-2024, it is important to act on two main axes: reduce the cesarean rate and strengthen postpartum monitoring. ^36^

## Conclusion

Maternal mortality rates remain high in Tunis. Most maternal deaths occurred postpartum after cesarean section. Hemorrhage continues to be the leading direct cause of maternal mortality, followed by COVID-19 infection.

### Ethical statement

This study was examined by the Ethics Review Committee on Maternity and Neonatology Center of Tunis on May 14, 2024. After assessment, the Ethics Review Committee has granted permission to proceed with the study’s conduct after the agreement of the regional health directorate of Tunis. The Ethics Review Committee accorded a waiver of consent participant because the research was conducted exclusively from the use of medical data from records (called data studies) while respecting the anonymity of patients. This study is not considered to be a study “involving the human person.” ^37^ We obtained the agreement of the director of the Regional Health Directorate of Tunis. The final decision of the Ethics Review Committee was obtained on August 14, 2024(Approval number: 15/2024). The confidentiality of data was respected, as was the anonymity of patients, health facilities, and healthcare staff.

### Consent to participate

Informed consent waiver was obtained by the Ethics Committee of Maternity and Neonatology Center of Tunis. The patients are deceased so we cannot have their consent. The study is not experimental on cadavers and not considered to be a study “involving the human person.” ^37^

The use of data from the files is permitted while respecting anonymity.

## Author contributions

Conceptualization, Khaoula Magdoud, Hanene Rezigui, Sirine Bayar, Hassine Saber Abouda and Rim Ben Hmid; Methodology, Khaoula Magdoud; Validation, Khaoula Magdoud, Hanene Rzigui, Najla Arifi, Sirine Bayar, Hamza Ben Abdallah, Hassine Saber Abouda and Rim Ben Hmid; Writing – original draft, Khaoula Magdoud, Hanene Rzigui, Najla Arifi, Sirine Bayar and Hamza Ben Abdallah.

## Data Availability

The data are not publicly available in accordance with institutional regulations of the Regional Health Directorate of Tunis. These data relate to deceased patients and are therefore easily identifiable by their ages, places of residence, their history and the circumstances of death. In case of request for scientific or research purposes, access to data is possible by asking the correspondent author Magdoud Khaoula ( khoula.magdoud@fmt.utm.tn). Harvard Dataverse: “Maternal Mortality in the Governorate of Tunis between 2017 and 2023: evolution of Maternal Mortality Ratio and the main causes of death” doi: https://doi.org/10.7910/DVN/9CEJ6R. This project contains the following:
• Questionnaire (in English and French) This work has been reported in line with the STROBE guidelines. ^38^ Harvard Dataverse: “Maternal Mortality in the Governorate of Tunis between 2017 and 2023: evolution of Maternal Mortality Ratio and the main causes of death” doi: https://doi.org/10.7910/DVN/9CEJ6R. ^39^ This project contains the following:
• STROBE-checklist form Data are available under the terms of the Creative Commons Zero “No rights reserved’ data waiver (CC0 1.0 Public domain dedication).
