# Supplementary material for: Maternal Mortality in the Governorate of Tunis between 2017 and 2023: evolution of Maternal Mortality Ratio and the main causes of death
Source: F1000Res. 2024 Oct 11;13:1218. [Version 1] doi: 10.12688/f1000research.155009.1 (PMC12547474; doi:10.12688/f1000research.155009.1)
Supplement: Peer review history

# Reviewer report: Reviewer response for version 1

**DOI:** 10.5256/f1000research.170122.r411706

**Published:** 2025-09-18

**Authors:** Samina Naeem Khalid, Junaid Sarfraz Khan

**Affiliations:** 1 MNCH (Maternal, Neonatal and Child Health) Department, Health Services Academy (Ringgold ID: 66692), Islamabad, Pakistan; 2 Public Health, Health Services Academy (Ringgold ID: 66692), Islamabad, Pakistan

**An Urgent Call from Tunis: Turning Maternal Mortality Data into Action**

**Prof. Dr. Samina Naeem Khalid**

 Email: saminanaeemkhalid@gmail.com, drsamina@hsa.edu.pk

**Prof. Dr. Junaid Sarfraz Khan**

 Email: junaidsarfraz@hsa.edu.pk

 The death of a woman due to pregnancy or childbirth is not just a statistic; it is a profound tragedy that exposes the very fabric of the health system's weaknesses in the country. The impact of a maternal death ripples outward, first devastating the lives of children, then destabilizing the family structure, and ultimately diminishing the progress of the entire nation. Today, with the medical knowledge and resources available, most of these deaths can be prevented, specifically by strengthening primary care (1).

 The recent study by Magdoud and colleagues in *F1000Research* shows that maternal mortality continues to pose a significant challenge in Tunisia’s capital region, highlighting critical concerns about equity, access, and quality of care (2). This study analyzed 61 maternal deaths in the Governorate of Tunis from 2017 to 2023, yielding findings that are both informative and insightful. An average MMR (Maternal Mortality Ratio) of 46.9 per 100,000 live births was observed, a figure more favorable than some regional benchmarks of the MENA (Middle East and North Africa) region but disproportionately high for a country with Tunisia’s healthcare resources (3). The peaks observed in 2017 and 2020 reflect underlying systemic weaknesses, and in 2020, the additional burden imposed by the COVID-19 pandemic surfaced as the second leading contributor, a stark reminder of how global crises place a disproportionate burden on pregnant women (4).

 Three priority areas for intervention emerged clearly from the study’s findings. The first concern is inequitable access to care, with almost 44% of deaths occurring among women referred from underserved regions outside Tunis. These delays are not simple logistical gaps; they are an injustice. No woman’s life should be put at risk because of where she happens to live. It is a stark reminder of the ‘three delays’ identified in the 1990s: seeking help, reaching a facility, and receiving care, that too often turn complications into tragedies (5). The shortage of obstetricians and intensive care facilities outside urban centers in Tunisia is part of a global inequity, one where rural women are left most vulnerable. According to the World Health Organization (WHO), unequal distribution of skilled health providers and emergency obstetric services remains a key contributor to maternal mortality, especially among rural and marginalized groups in low and middle-income countries (6).

 The second issue is the high prevalence of cesarean sections, as the national cesarean section rate in Tunisia surpassed 43% in 2018, which is quite concerning. This heavy reliance on surgical delivery carries serious risks that can no longer be overlooked, as 87.5% of postpartum deaths occurred following cesarean deliveries. The findings demand urgent scrutiny of obstetric practices, clinical decision-making, and the systemic forces fueling high cesarean rates. Cesarean sections are undeniably life-saving when medically indicated, but their overuse is dangerous, a concern highlighted by the WHO (7).

 Third, analysis of the evolving causes indicates that the hemorrhage persists as the predominant direct cause of maternal mortality, reflecting a global challenge that has shown limited improvement over time. The WHO’s 2023 global report on maternal mortality also highlights the unfinished agenda of addressing preventable causes of hemorrhage and hypertensive disorders. This underscores the urgent need to ensure that pregnant women are central to emergency preparedness and public health strategies (8).

 While Tunisia’s MMR of 46.9 per 100,000 live births remains concerning for the country, it is significantly lower than Pakistan’s national average of 186 per 100,000, as documented in the Pakistan Maternal Mortality Survey 2019 (9). Pakistan bears one of the heaviest maternal mortality burdens in South Asia, driven by systemic gaps in emergency obstetric care, limited access to skilled birth attendants, and significant rural and urban disparities. The study by Midhet et al. (2025) provides a valuable long-term perspective on maternal mortality in Pakistan, showing both the persistent burden and the changing pattern of risk factors over the past decade. Their findings are consistent with Magdoud et al. (2024), showing that even moderate national MMRs can mask severe underlying inequities. This is also critically relevant in Pakistan, where structural barriers, economic hardship, and gender inequality disproportionately endanger women (10). Thus, Tunisia's experience demonstrates that reducing overall maternal mortality is not enough unless efforts also focus on addressing disparities and ensuring that neither geography nor poverty determines a woman’s chance of survival.

 According to Kumari et al. (2025), global maternal mortality has shown a consistent decline over time, largely driven by stronger health systems, expanded access to skilled birth attendants, and significant progress in obstetric care. However, the findings of Magdoud et al. (2024) highlight that global gains may mask enduring local challenges (11).

 A recent study by Nist (2025) emphasizes that, despite overall declines, maternal mortality persists as a critical global health concern, with preventable causes still claiming women’s lives, especially in low- and middle-income countries (12). The report emphasizes the urgent need to ensure equitable access to skilled care, prompt emergency obstetric services, and robust health systems across all settings, reinforced by findings of Magdoud et al. (2024) as a case study.

 Dehghani and colleagues (2025) argue that maternal deaths globally are driven by a consistent pattern of clinical causes like hemorrhage and hypertensive disorders, exacerbated by systemic delays. This pattern is clearly illustrated by the findings from Tunisia, reinforcing the urgent need for coordinated interventions, equitable resource distribution, and stronger accountability mechanisms to address these universal failures (13). These two studies show how local surveillance and cause-of-death analyses can inform global strategies, ensuring that efforts to reduce maternal mortality stay relevant to specific contexts while aligning with international frameworks.

 The findings of Magdoud et al. function as both a stark warning and a potential roadmap for interventions, demonstrating that a striking 69% of maternal deaths were attributable to preventable causes. Their work reminds us that behind every maternal death, a sentinel event, lies a cascade of systemic failures that abandon women in their most vulnerable moments. Tunisia has made significant progress, yet the work is still not finished. As the world aims to reach the Sustainable Development Goal of reducing maternal mortality to fewer than 70 deaths per 100,000 live births by 2030, Tunis sends a clear message that achieving real progress requires not only clinical excellence but also equity, accountability, and compassion. The women of Tunisia and women across the world deserve care that is equitable, respectful, and capable of saving lives. The challenge now is to turn evidence into policy and practice so that no woman dies while giving life.

1. **Mayrink, J., Costa, M. L., Souza, R. T., Sampaio, L. T., & Cecatti, J. G. (2025). **Prevention of maternal mortality with interventions in primary care services: What can we do?. International Journal of Gynecology & Obstetrics, 169(1), 81-88.
2. **Magdoud, K., Rezigui, H., Arifi, N., Bayar, S., Ben Abdallah, H., Abouda, H. S., & Ben Hmid, R. (2024). **Maternal Mortality in the Governorate of Tunis between 2017 and 2023: evolution of Maternal Mortality Ratio and the main causes of death. *F1000Research*, *13*, 1218.
3. **Moujahid, C., Turman, J. E., & Amahdar, L. (2024). **A scoping review of the social determinants of maternal health in the MENA region. *Pan African Medical Journal*, *47*(1).
4. **BOUNOUH, A. (2021).** The devastating impact of COVID-19 on the health system in Tunisia. *Moroccan Journal of Public Health*, *2*(1), 22-44.
5. **Thaddeus, S., & Maine, D. (1994). **Too far to walk: maternal mortality in context. *Social science & medicine*, *38*(8), 1091-1110.
6. **Glover, A. L., Brown, D., Holman, C., & Nelson, M. (2025). **Obstetric care in rural critical access hospitals: A domestic application of the World Health Organization Emergency Obstetric Care framework in rural communities. The Journal of Rural Health, 41(2), e70037.
7. **Opiyo, N., Kingdon, C., Oladapo, O. T., Souza, J. P., Vogel, J. P., Bonet, M., ... & Betrán, A. P. (2019). **Non-clinical interventions to reduce unnecessary caesarean sections: WHO recommendations. *Bulletin of the World Health Organization*, *98*(1), 66.
8. **World Health Organization. (2023).** *Trends in maternal mortality 2000 to 2020: estimates by WHO, UNICEF, UNFPA, World Bank Group and UNDESA/Population Division*. World Health Organization
9. **National Institute of Population Studies-NIPS**. Pakistan Maternal Mortality Survey. Islamabad/Pakistan: NIPS/ICF, 2020. (Available from:

https://nips.org.pk/publication/pakistan-maternal-mortality-survey-pmm

1. **Midhet, F., Khalid, S. N., Baqai, S., & Khan, S. A. (2025). **Trends in the levels, causes, and risk factors of maternal mortality in Pakistan: A comparative analysis of national surveys of 2007 and 2019. *Plos one*, *20*(1), e0311730.
2. **Kumari, C. K., Anuragamayi, Y., Gundu, V., & Ritika, M. **(2025). Declining trends in Maternal Mortality Rate. *European Journal of Cardiovascular Medicine*, *15*, 397-402.
3. **Nist, M. D.** (2025). Global Maternal Mortality. *MCN: The American Journal of Maternal/Child Nursing*, *50*(5), 307.
4. **Dehghani, N., Naseri, M., & Kharazmi, H. A.** (2025). Reducing Maternal and Infant Mortality: Global Challenges and Integrated Strategies Toward 2030. *Health Providers*, *5*(1), 135-140.

Is the work clearly and accurately presented and does it cite the current literature?

Yes

If applicable, is the statistical analysis and its interpretation appropriate?

Yes

Are all the source data underlying the results available to ensure full reproducibility?

Yes

Is the study design appropriate and is the work technically sound?

Yes

Are the conclusions drawn adequately supported by the results?

Yes

Are sufficient details of methods and analysis provided to allow replication by others?

Yes

Reviewer Expertise:

Dr. Samina Naeem Khalid, MBBS, MPhil, PhD, MSPH, CHPE, Fellowship ReproMed (USA), is Head of the Maternal, Neonatal, and Child Health Department (MNCH) at the Health Services Academy, Islamabad. She is a Health Expert with over 30 years of experience in clinical practice, teaching, research, and policy advocacy. She has collaborated with the Ministry of Health, WHO, UNICEF, USAID, UNFPA, and other international partners on reproductive health, family planning, cervical cancer prevention, and safe motherhood initiatives. Dr. Khalid holds advanced training in reproductive medicine and infertility (IVF/ICSI) from the Cleveland Clinic, USA, and has published extensively on maternal mortality, unsafe abortions, and HPV vaccine introduction in Pakistan. A passionate advocate for women’s health, she has trained hundreds of healthcare providers, supervised over 50 postgraduate students, and led national campaigns such as What Women Want, amplifying the voices of more than 250,000 women and girls.

We confirm that we have read this submission and believe that we have an appropriate level of expertise to confirm that it is of an acceptable scientific standard.

# Reviewer report: Reviewer response for version 1

**DOI:** 10.5256/f1000research.170122.r366690

**Published:** 2025-02-26

**Authors:** Roger Smith

**Affiliations:** 1 Mothers and Babies Research Centre, Hunter Medical Research Institute, University of Newcastle, Newcastle, Australia

This paper provides a summary of recent maternal mortality in Tunis. It is well written and highlights the problem of transport from regional centres to the obstetric care facilities in Tunis. It also highlights the high rates of caesarean section. It does not suggest solutions for regional women to promptly obtain care in the setting of post-partum haemorrhage. Is it possible for the military to provide helicopter evacuation as has been described in Nepal (Sharma et al., AJOG).

Is the work clearly and accurately presented and does it cite the current literature?

Yes

If applicable, is the statistical analysis and its interpretation appropriate?

Yes

Are all the source data underlying the results available to ensure full reproducibility?

Yes

Is the study design appropriate and is the work technically sound?

Yes

Are the conclusions drawn adequately supported by the results?

Yes

Are sufficient details of methods and analysis provided to allow replication by others?

Yes

Reviewer Expertise:

Maternal Mortality

I confirm that I have read this submission and believe that I have an appropriate level of expertise to confirm that it is of an acceptable scientific standard.
